# An atlas of the germ ball-cercaria-schistosomulum transition in *Schistosoma mansoni*, using confocal microscopy and *in situ* hybridisation

**DOI:** 10.1016/j.crpvbd.2022.100087

**Published:** 2022-04-12

**Authors:** Sophie J. Parker-Manuel, R. Alan Wilson

**Affiliations:** a Department of Biology, University of York, Heslington, York YO10 5DD, UK; b York Biomedical Research Institute, University of York, Heslington, York YO10 5DD, UK

**Keywords:** *Schistosoma mansoni*, Development, Myogenesis, Sm16 stathmin, SmKK7, Peripheral nerve net, Infection process

## Abstract

Schistosomes are complex platyhelminth parasites with a genome comprising ∼12,000 protein-coding genes, three distinct generations, and at least seven distinct phenotypes. We chart here cellular and gene expression changes associated with development of the cercaria, in the intramolluscan daughter sporocyst, and its transformation into the skin stage schistosomulum upon infection of the mammalian host. We describe the morphology of the early daughter sporocyst and the increasing complexity of cellular organisation in germ balls as they rapidly develop into cercariae. We show how individual myocytes differentiate and combine to create the complex musculature of the head capsule and body wall. *In situ* hybridisation reveals that some transcripts encoding the secretory proteins, released during skin penetration, are expressed in gland-cell precursors very early in germ ball development. However, those for the projected anti-inflammatory protein Sm16-stathmin are widely expressed in germ ball tissues, suggesting the protein has intracellular functions. Transcripts for *smkk7* are expressed in six cells of the larval body, while the KK7 protein is present throughout the peripheral nerve net, including sensory nerve bulbs, providing a marker for the nerve net in adult worms. We also note that the cercaria-schistosomulum transformation is accompanied by tissue remodelling without growth.

## Highlights

• Vermiform daughter sporocysts are imaged *en route* to the hepatopancreas.
• Morphology of germ ball development into the mature cercaria is charted.
• Developing musculature of head capsule and body wall is visualised with phalloidin.
• Distribution of SmKK7 protein highlights the layout of the peripheral nerve net.
• *In situ* hybridisation shows widespread expression of Sm16 in germ ball tissues.

## Graphical abstract

**Figure:**
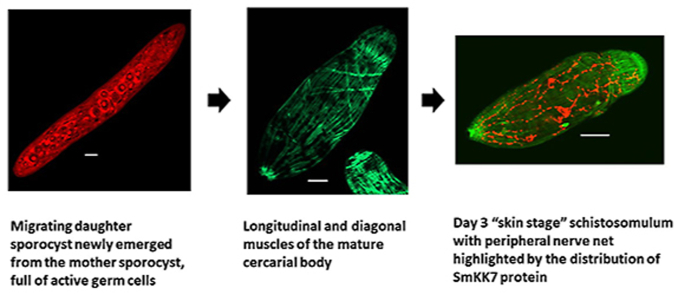

## 1 Introduction

**Fig. 1:**
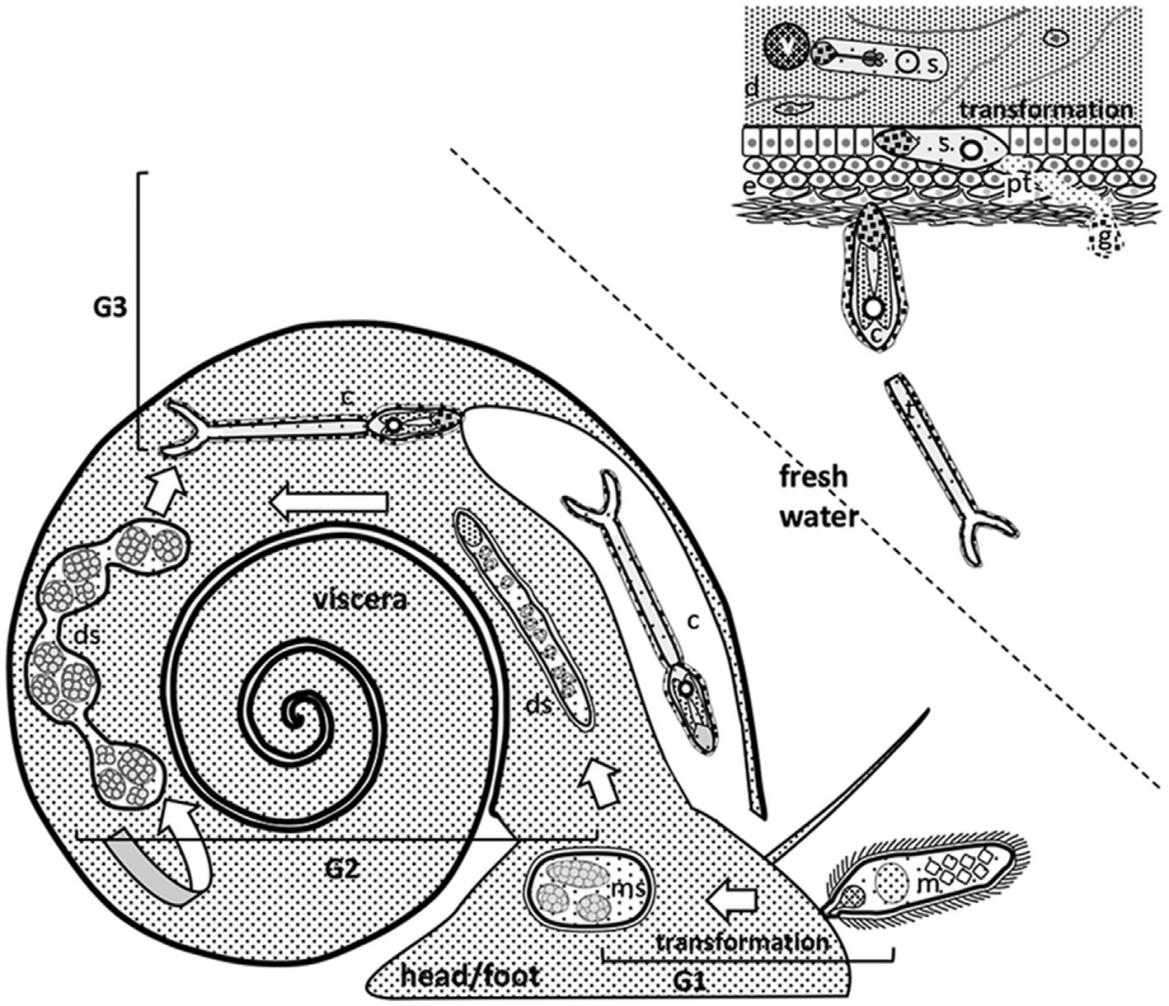
Schematic illustration of the life-cycle generations associated with the snail, and infection of the mammalian host (not to scale). G1: The planorbid snail is infected by a miracidium larva, newly hatched from a mature egg deposited in freshwater. As it penetrates the head/foot, the miracidium sheds its ciliated epithelial plates and transforms into the mother sporocyst, which remains close to the penetration site. Germinal cells within the mother sporocyst develop into the next generation daughter sporocyst. G2: The daughter sporocyst emerging from the mother sporocyst, contains individual large germinal cells. It is equipped to migrate through the snail haemocoel from the head/foot to the viscera, housed within the spirals of the shell. Here it enlarges and develops discrete brood chambers where germ balls develop. These may give rise to further G2 daughter sporocysts, or to the third generation, represented by the cercaria larva. G3: The cercaria leaves the daughter sporocyst and travels through the haemocoel to the mantle region where it exits to freshwater. It is non-feeding, relying on glycogen stores to maintain swimming activity, while locating the mammalian host. It infects by direct penetration of the skin, using the proteases of its acetabular gland-cells, at the same time losing its propulsive tail and shedding its osmo-protective glycocalyx. It rapidly reaches the base of the epidermis where transformation to the schistosomulum takes place. This involves tissue remodelling, with replacement of the tegument surface by a new membrane configuration, and resorption of acetabular gland-cells. The schistosomulum then traverses the dermis and exits *via* a venule, using the secretions of its head gland. *Abbreviations*: G, generation; c, cercaria; d, dermis; ds, daughter sporocyst; e, epidermis; g, glycocalyx; m, miracidium; ms, mother sporocyst; pt, penetration tunnel; s, schistosomulum; t, tail; v, venule.

Schistosomiasis, caused by blood flukes of the genus *Schistosoma*, remains a serious parasitic disease, causing morbidity and death. Its transmission is linked to water contact and a lack of sanitation (McManus et al., 2018). A single drug, praziquantel, is used in mass drug administration programmes to control the disease, and no vaccine is available; in the absence of acquired immunity, individuals require frequent drug treatment due to reinfection. *Schistosoma mansoni* has a two host life-cycle, with an aquatic gastropod mollusc intermediate host which becomes infected when a miracidium larva penetrates the head/foot, where it transforms into the mother sporocyst in proximity to the penetration site (Yoshino et al., 2016). The developmental stages within the snail, the emergence of the cercaria larva into fresh water, its penetration into mammalian (human) host skin, and subsequent transformation into the schistosomulum are summarised in Fig. 1.

Development of germ balls within the daughter sporocyst has been described (e.g. Ebrahimzadeh, 1970; Hockley, 1972; Cheng & Bier, 1972; Dorsey, 1975). A ball starts out as a small spherical mass of cells, which enlarges before the posterior flattens and buds the tail; this bifurcates and extends in parallel with development of the internal body tissues. A time-base for the process is lacking but pulse-incubation of infected snails with ^75^Se methionine revealed that relevant proteins are synthesised in developing germ balls 9 to 5 days before mature cercariae emerge from the snail (Wilson & Coulson, 1986). This indicates that to capture expression of transcripts encoding the proteins necessary for infection, germ balls need to be included in analyses of gene expression patterns (Parker-Manuel et al., 2011). Mature cercariae have been comprehensively studied at the ultrastructural level (Dorsey et al., 2002) and an atlas of cercarial tissues has been compiled using specific cell-type markers, coupled with confocal microscopy (Collins et al., 2011). Of particular note are six pre-and four post-acetabular gland-cells, the secretions from which mediate skin penetration (Hackey & Stirewalt, 1956), and a complex array of sensory endings in the apical area, which co-ordinate the process (Nuttman, 1971; Dorsey et al., 2002). The transition from aquatic cercaria to tissue-dwelling schistosomulum is accompanied by profound changes in physiology and morphology, including loss of the propulsive tail and osmo-protective glycocalyx. Tissue remodelling begins almost immediately with the acquisition of a new tegument surface complex (Hockley & McLaren, 1973).

This mammalian infection process has been investigated at both the transcriptomic and proteomic levels. A genome-wide microarray analysis of gene expression in germ balls, cercariae and day 3 schistosomula identified a large number of proteases that may be involved in skin invasion, venom allergen-like (VAL) and micro-exon gene (MEG)-encoded proteins. The VALs are mostly enriched in the germ ball, whereas the MEGs are strikingly upregulated in the schistosomulum (Parker-Manuel et al., 2011). Analysis of the proteins released during cercaria-schistosomulum transformation by proteomics identified both previously known cercarial elastase (Newport et al., 1988) and Sm16 (Rao & Ramaswamy, 2000), plus novel invadolysin, three VAL proteins, dipeptidyl peptidase IV, and SmKK7. This last was designated as ‘KK7’ on the basis of homology with a venom protein from the scorpion *Mesobuthus martensii* (Curwen et al., 2006).

The present work attempts to link the morphological changes in the transition from developing germ ball through cercaria to the skin stage schistosomulum, with the gene and protein expression patterns over the same period. We use whole mount *in situ* hybridisation (WISH) here for the first time with germ balls, previously applied to schistosomula and adult schistosomes (Dillon et al., 2007; DeMarco et al., 2010; Cogswell et al., 2011), to chart the expression pattern in time and space of genes encoding proteins involved in the infection/transformation process. Parallel immunocytochemistry is also used to localise protein expression in larval tissues. Notably, unexpected patterns for Sm16 and SmKK7 expression were found, leading to new hypotheses about the possible functions of these proteins.

## 2 Methods

### 2.1 Biological material

All parasite material was from a Puerto Rican isolate maintained at the University of York, UK, by passage through in-house bred NMRI mice and albino *Biomphalaria glabrata* snails. Germ balls, cercariae and schistosomula were obtained as previously described (Parker-Manuel et al., 2011). Daughter sporocysts and germ balls were harvested separately using a clean drawn-out glass pipette and kept in filter sterilised 50% PBS on ice until use. Adult worms were obtained by portal perfusion of mice up to 7 weeks post-infection with 180 cercariae.

### 2.2 Langeron’s carmine staining

Parasites were fixed in AFA (22% formalin, 33% 95% ethanol, 11.5% glacial acetic acid, 33.5% water) overnight, and then stored in 70% ethanol until use (at least 10 min if they were used immediately). The ethanol was removed and replaced with a few drops of the protein stain Langeron’s carmine (LC) and incubated at room temperature for 50 min. Excess stain was removed by washing the parasites several times with 70% acid ethanol. The parasites were then dehydrated through 90% ethanol and two changes of absolute ethanol (10-min incubations at room temperature and pressure (RTP)). They were cleared for 15 min in Histoclear (Fisher Chemicals, Loughborough, UK), mounted onto slides with DPX (VWR Chemicals, Poole, Dorset, UK), allowed to dry overnight and stored at room temperature*.*

### 2.3 Phalloidin and DAPI (4′,6-diamidino-2-phenylindole) staining

Daughter sporocysts, germ balls, cercariae and skin stage schistosomula were stained with Alexafluor 488-conjugated phalloidin (Invitrogen, Molecular Probes, Waltham, MA, USA) to visualize f-actin, and counterstained with DAPI according to Bahia et al. (2006). Specimens were fixed for one hour in 4% paraformaldehyde in PBS on ice and then permeabilised for an hour in PBS, 0.2% gelatin, 0.1% saponin, 0.1% NaN_3_ (PGN) at room temperature, before addition of phalloidin (0.0165 μM, 20 min) and/or DAPI (0.01 μM, 30 min) in PGN at room temperature. They were washed several times in PBS before they were viewed with a Zeiss inverted confocal microscope.

### 2.4 Confocal microscopy

Confocal microscopy was carried out using a Zeiss LSM 510 meta on an Axioplan 2M (upright), a Zeiss LSM 510 meta on an Axiovert 200M (inverted), or a Zeiss LSM 780 multiphoton for whole mount adult worms. Images were usually acquired with a Plan-Aprochromat 63×/1.4 oil DIC under oil immersion. For Langeron’s carmine a 543 nm laser was used with an HFT 488/543 beam splitter and a long-pass 585 nm filter. Microscope settings for phalloidin staining were 488 nm laser excitation, with a 405/488/543 nm beam splitter and a Band Pass 505–530 nm filter. For DAPI, a 405 nm laser was used with a 405/488/543 nm beam splitter and a 420–480 nm band pass filter. Propidium iodide (PI)-stained specimens were viewed using a 543 nm laser with a long-pass 585 nm filter.

### 2.5 Probe synthesis for whole mount *in situ* hybridisation (WISH)

RNA was extracted from germ balls using TRIzol (ThermoFisher Scientific, Swindon, UK) according to the manufacturer’s instructions. The samples were quality assessed using a Nanodrop spectrophotometer (ThermoFisher Scientific, Swindon, UK) and Bioanalyser picochip (Agilent, Santa Clara, CA, USA). A 2 μg sample of RNA was reverse-transcribed in a 20 μl reaction using Omniscript RT (Qiagen, Hilden, Germany) with an oligo dT primer (Promega, Chilworth, Hampshire, UK). Genes chosen for WISH were amplified by PCR with the following primers designed using sequences available at WormBase ParaSite (https://parasite.wormbase.org/species.html): Cercarial elastase 1a (Smp_119130) (forward: 5′-CTG TCA TCG CAT TCT TAA CGA C-3′; reverse: 5′-CGT TAT CAT CCC TTC CAT AAC C-3′); Invadolysin (Smp_090100) (forward: 5′-ATG ATA CCC TGT TCA AGA AAT CTC TT-3′; reverse: 5′-CCT ATC AGT TGT AGG ATG CAT TTC-3′); *Sm16* (Smp_113760) (forward: 5′-ATG ACA TTG ATC ACA GCT ACA ACG-3′; reverse: 5′-CAT CAT CTT ATC CAG TTT CTT CGC-3′); *SmKK7* (Smp_194830) (forward: 5′-GAA TTC AAA CCT GGC CGA GTC AAG TGC AGC G-3′; reverse: 5′-TCT AGA TCA TGC AAT TTA TGT TCA TCA TAG G-3′); *val 10* (Smp_002060) was subcloned from pENTR using primers to the vector (forward: 5′-GCC TTG TTT AAC TTT AAG AAG GAG C-3′; reverse: 5′-ATA ATG ACT TTG TAC AAG AAA GC-3′); *meg3.2* (Smp_138070) (forward: 5′-TTA ATT ATA GTT AAC AAA CAG CCA AGA-3′; reverse: 5′-TCG ACT GTG TAT TCA CAG CTC G-3′); *Sm29* (Smp_072190) (forward (5′-ATG TGA ATG TGT ATG GAA GAA GGT AG-3′; reverse: 5′-TTT CTC GGA ATT GAA GTC GC-3′).

PCR products were ligated into PGEM-T Easy (Promega, Chilworth, Hampshire, UK) according to the manufacturer’s instructions. A 2 μl aliquot of the resulting ligation was transformed into chemically competent DH5α *Escherichia coli* (Invitrogen, Waltham, MA, USA). The bacteria were spread onto LB agar plates with 0.1 mg/ml ampicillin and 40 μg/ml X-gal for blue/white selection. White colonies were picked and grown in 10 ml LB cultures with 40 μg/ml ampicillin overnight at 37 °C with shaking. Plasmid DNA was extracted using a Qiagen miniprep kit according to the manufacturer’s instructions, then sequenced by the in-house Technology Facility using T7 primer to check the quality and direction of the insert. Sequences were checked by BLAST analysis against the *S. mansoni* genome at www.genedb.org using the default settings. Digoxigenin (DIG)-labelled probes were synthesised using the method described by Dillon et al. (2007).

### 2.6 Whole mount *in situ* hybridisation

WISH was carried out on cercariae, schistosomula, and adults according to the method described by Dillon et al. (2007). However, the more delicate germ balls do not withstand proteinase K treatment; they were permeabilised in methanol and treated according to the following method. First, they were fixed in 4% paraformaldehyde at 4 °C overnight with shaking, then washed twice for 5 min in PBS and stored in PBS at 4 °C. Permeabilisation and dehydration was carried out through 10-min washes in a graded series of PBS with 0.1% Tween 20 (PBSAT) plus 25%, 50% and 75% MeOH at room temperature, before two washes in 100% MeOH. Parasites were stored in 100% MeOH at −20 °C until use, or for at least 30 min if used immediately. They were rehydrated through 10-min washes at room temperature in a graded series 100%, 75%, 50% and 25% MeOH in PBSAT, followed by two washes in PBSAT. Following rehydration, the samples were incubated in pre-hybridisation solution (50% formamide, 5× saline sodium citrate buffer (SSC, pH7), 2% blocking powder (Roche Diagnostics, Burgess Hill, UK), 1% Triton X-100, 0.5% CHAPS, 100 μg Yeast RNA, 50 μM EDTA, and 50 μg/ml heparin) for 1 h at 65 °C. After this step the prehybridisation buffer was replaced with fresh hybridisation solution containing 2 μl/ml digoxygenin (DIG)-labelled RNA probe and the germ balls were incubated overnight at 65 °C with constant rocking.

Hybridisation solution was removed, and specimens were washed twice 30 min each in pre-warmed Solution 1 (50% formamide, 5× SSC pH 4.5, 1% SDS) at 65 °C, followed by two 30-min washes in Solution 2 (50% formamide, 2× SSC pH 4.5, 1% Tween 20) at 65 °C. Next, the parasites were washed three times in tris-buffered saline and Tween (TBST; 0.14 M NaCl, 2.7 mM KCl, 25 mM Tris-HCl pH 7.5, 0.1% Tween) for 5 min each at room temperature. This was followed by a blocking step: 90-min incubation at room temperature in TBST with 10% heat-inactivated sheep serum. Germ balls were incubated in fresh blocking solution containing alkaline phosphatase-conjugated anti-DIG FAb fragments (Roche Diagnostics, Burgess Hill, UK) (1:2000 dilution) overnight at 4 °C. Antibody solution was removed, and the germ balls were subjected to three 5-min washes, and four 1-h washes in TBST at room temperature. A final TBST wash was applied overnight at 4 °C. Colour was developed by addition of BM purple (Roche Diagnostics, Burgess Hill, UK) or Fast Red fluorescent substrate (Sigma, Gillingham, Dorset, UK). When signal had developed sufficiently, germ balls were washed twice in PBSAT and stored in 10% formalin in PBSAT. Negative controls were always included, using DIG-labelled sense probe for the *S. mansoni* chorion gene. Parasites were imaged using a Leica DM2500 microscope attached to an 18.2 colour mosaic camera (Diagnostic Instruments, Sterling Heights, MI, USA) with SPOT Advanced software (SPOT Imaging Solutions, Sterling Heights, MI, USA).

### 2.7 Immunocytochemistry (ICC)

Larvae were fixed overnight in 4% paraformaldehyde at 4 °C with shaking. They were then washed twice for at least 5 min in PBS and stored at 4 °C until use. All the subsequent steps were carried out at 4 °C with shaking. Bodies were washed three times in permeablising fluid (PBS, 1% triton X-100, 0.1% SDS, 10% naïve sera from secondary antibody species, 0.1% NaN_3_), 30 min. This fluid was replaced with antibody diluent (AbD) PBS with 0.5% Triton X-100, 0.1% BSA, 10% naïve sera from secondary antibody species, 0.1% NaN_3_ and 1:200 antiserum to recombinant antigen. Parasites were incubated for 4 days at 4 °C with shaking. Non-specifically bound antisera were removed by 3 washes in AbD over 24 h at 4 °C with shaking. Secondary goat anti-rat antibody was added at 1:200 in AbD and incubated for 48 h. Whole mount ICC was carried out on adult worms according to the protocol described by Mair et al. (2000). For adults, the secondary antibody was conjugated to Alexafluor 488, but for larvae the fluorophore was Alexafluor 647 (Invitrogen Waltham, MA, USA). Excess secondary antibody was removed by 3–4 washes in AbD over 24 h. Larvae were counterstained with Alexafluor 488-conjugated phalloidin by incubating for 2–3 days in AbD. After two brief washes in AbD, larvae were mounted on slides and viewed with a confocal microscope.

To investigate the distribution of SmKK7 in adults ICC was also carried out on cryosections of males as previously described (Li et al., 2014). Briefly, the worms were fixed for 4 h in 4% formaldehyde in phosphate-buffered saline (PBS), washed in PBS, before being transferred to optimum cutting temperature (OCT) compound and frozen on dry ice. Seven-micron sections were cut and reacted with rat anti-SmKK7 antiserum, before being rinsed and incubated with Alexafluor 488-labelled goat anti-rat antibodies (Invitrogen, Waltham, MA, USA). The sections were viewed by confocal microscopy as above.

### 2.8 Electron microscopy

Adult male schistosomes were fixed in 4% formaldehyde in PBS at room temperature and processed for scanning electron microscopy (SEM) as previously described (Li et al., 2014) to observe the tegument surface.

### 2.9 Reproducibility information

Approximately 100 replicates of each stage were processed for the WISH, 20–50 for ICC and LC/phalloidin, and 10–20 adults for the WISH and ICC. The unusual pattern of WISH staining for *sm16* was confirmed by recloning and repeating the WISH multiple times.

## 3 Results

### 3.1 Daughter sporocysts

**Fig. 2:**
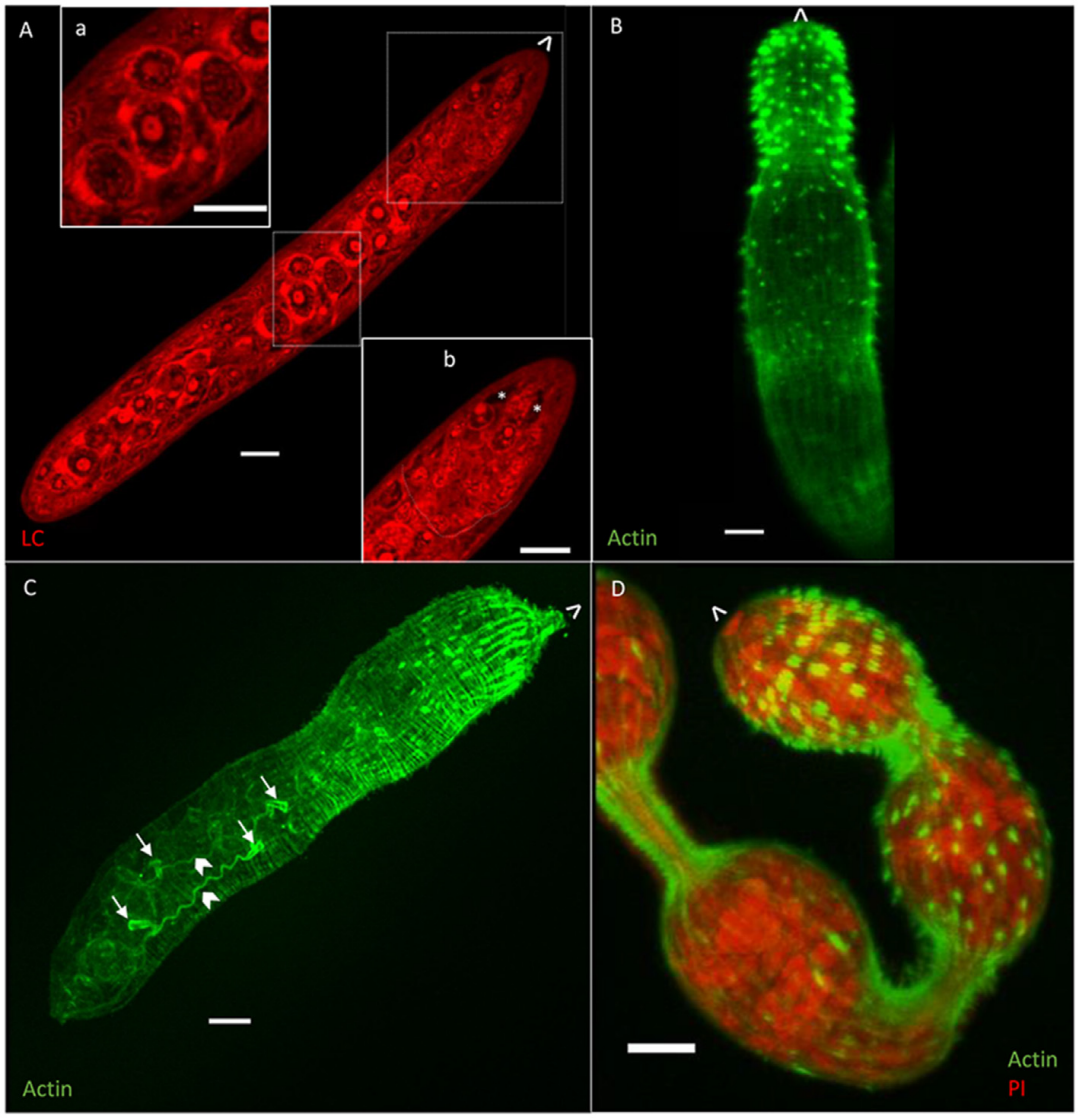
Confocal images of daughter sporocysts. The anterior of the parasite is indicated by ˆ. **A** Single confocal image of a vermiform daughter sporocyst stained with Langeron’s carmine. Insets show the large germ cells and the anterior of the parasite. Potential gland-cells indicated (∗). **B** A vermiform specimen stained with phalloidin. **C** Projection of a z-stack of a phalloidin-stained specimen. Flame cells (arrows) and their tubules (chevrons) are visible. **D** Projection of a z-stack showing a maturing daughter sporocyst stained with phalloidin and propidium iodide (PI). The anterior of the parasite is indicated by ˆ. *Scale-bars*: 10 μm.

Development of larval schistosomes in the snail is asynchronous, with daughter sporocysts emerging from the mother sporocyst over a period of days to weeks. Here we show a snapshot of daughter sporocysts collected from the haemolymph/hepatopancreas of snails at day 21 post-infection. During this migratory phase they are all the same size and morphology (*n* > 10). A vermiform migratory stage specimen stained with Langeron’s carmine (180 μm long, 26 μm wide) has a thin body wall (1.5–2.0 μm) enclosing numerous irregularly shaped cells (Fig. 2A). The outermost smaller cells (4–5 μm in diameter) surround fewer large cells (8–10 μm in diameter) that comprise the central core of the body. These core cells are almost certainly the germinal cells, which will divide to develop into germ balls before either developing into daughter sporocysts or maturing into cercariae. A putative germinal cell (Fig. 2A, inset a) viewed in more detail, has a large nucleus (dotted white outline, 9 μm at its widest point) containing condensed chromatin (stained red) and a very large nucleolus (white outline, 3.2 μm in diameter). Intense staining of the cytosol indicates protein synthesis is underway. The anterior of the larva lacks obvious germinal cells (Fig. 2A, inset b), but there is a clear posterior demarcation, which the entire z-stack reveals is not phalloidin-positive, hence not composed of actin filaments (cf. the cercarial head capsule). Within this region, two unstained narrow elongated structures are visible at the very anterior (Fig. 2A, asterisks), reminiscent of the head gland in the cercaria.

Phalloidin staining reveals that the anterior end of the vermiform daughter sporocyst is covered with posterior-facing, actin-rich spines (Fig. 2B). Spine distribution is sparse in the mid-region of the sporocyst and absent at the posterior end. Numerous tightly packed and narrow circular muscle fibres are evident, forming the outer layer of the body wall (Fig. 2C), beneath which are thicker but less frequent longitudinal fibres. There are four flame cells (Fig. 2C, arrows) within the body core, along with their collecting tubules (Fig. 2C, chevrons). As the daughter sporocyst develops further, it loses its vermiform appearance (Fig. 2D) and bulbous regions of the body become apparent, connected by actin-rich isthmuses, where circular muscle fibres are contracted. The surface spines are still evident, with the same distribution as in the vermiform specimens, most numerous on the most anterior bulge where they are paired, with fewer visible on the second bulge. These spheroidal bulges are the ‘brood chambers’, which PI staining reveals contain many cell clusters representing several developing germ balls that will differentiate either into daughter sporocysts or cercariae.

### 3.2 Germ ball development revealed by Langeron’s carmine staining

**Fig. 3:**
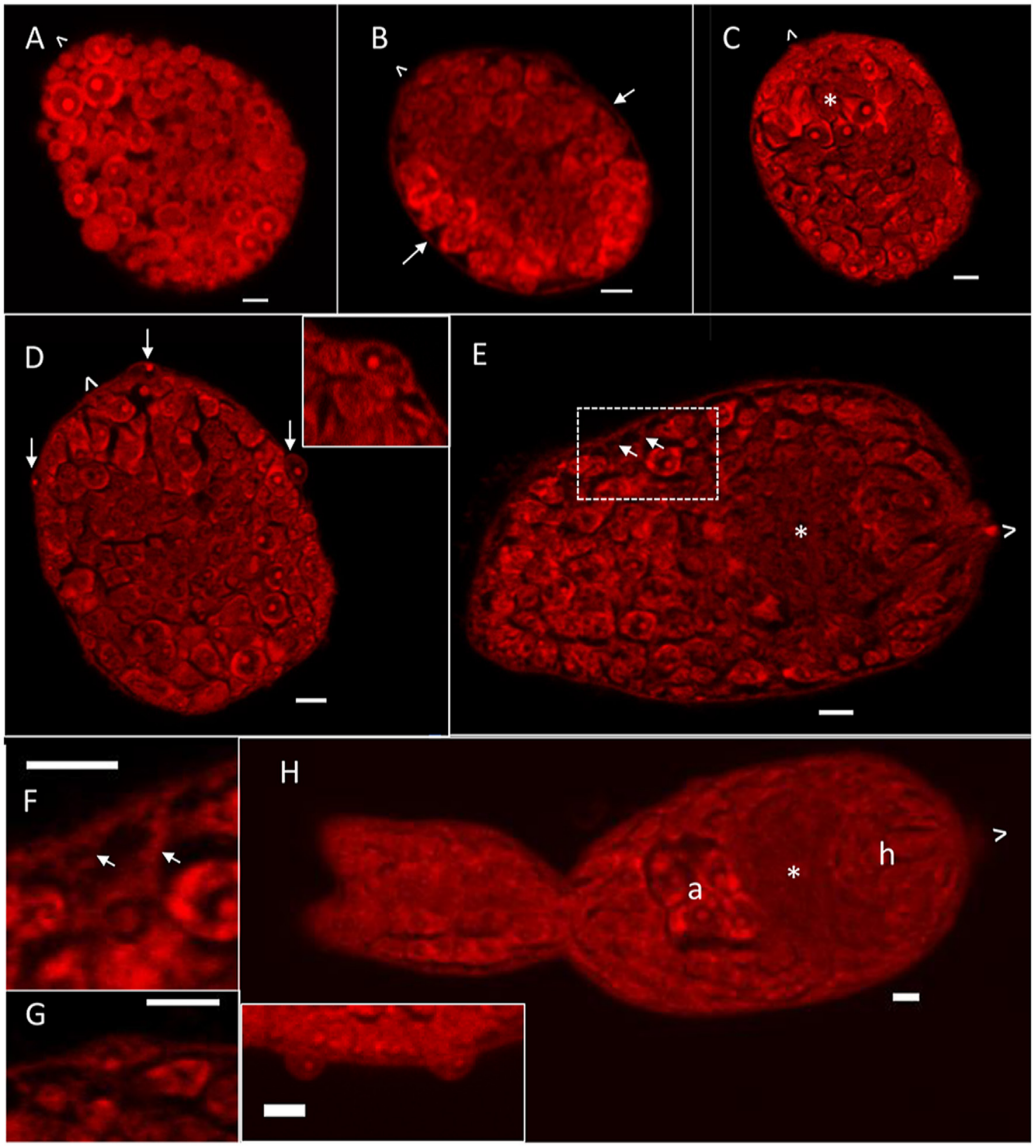
Germ ball development revealed by Langeron’s carmine staining. **A** Early germ ball composed of > 100 loosely packed spherical cells. **B** Germ ball enveloped by a visible thin epithelial layer. **C** and **D** are optical sections from the same z-stack. **C** Flat-bottomed germ ball with cells beginning to organise. Asterisk indicates cells arranged in an arc towards the anterior. **D** Epithelial nuclei are visible at the surface (arrows). Inset: enlargement of cells contributing to the outer epithelial layer. **E** An elongated germ ball with cells organising into tissues. Asterisk indicates developing neural ganglia. **F** and **G** are enlarged areas from the z-stack of the specimen in **E**. **F** is the inset showing connections between the surface and internal cells. **G** is from a different layer in the stack showing a remaining surface cell. The connections are indicated by arrows in **E** and **F**. **H** ‘Stubby-tailedʼ germ ball with developing head capsule (h), neural ganglia (∗) and acetabular gland-cells (a). Inset: enlarged section from the tail surface showing remaining surface cells. The anterior of the parasite is indicated by ˆ. *Scale-bars*: 5 μm.

Germ ball development is asynchronous, with several stages present in each daughter sporocyst at any one time. The terminology used for description is that of Cheng & Bier (1972). As a rule, the smaller the germ ball, the less developed it is. Each early-stage germ ball (∼57 μm) consists almost entirely of roughly spherical and loosely aggregated cells (Fig. 2A), which vary in diameter from 2 to 9 μm, with prominent nucleoli and a peripheral brightly staining ring of cytoplasm. A bounding membrane is imperceptible at this stage. At the next morphological stage (∼45 μm long), a thin layer of cytoplasm, which will become the tegument, envelops the entire germ ball (Fig. 3B, arrows). The no-longer spherical cells are more closely apposed and appear to be organising themselves into structures (55 μm long and 45 μm wide; Fig. 3C), while the thin surface layer becomes more apparent as an epithelium (possibly a syncytium) with visible nuclei (Fig. 3D, arrows and inset). Towards the anterior of the germ ball a group of cells is arranged in an arc (Fig. 3C, asterisk), as the posterior end becomes flattened.

When the germ ball is approximately 75 μm in length (Fig. 3E) the enveloping epithelium/syncytium and some contributing nuclei are still clearly visible (Fig. 3G) but additionally, bridges of cytoplasm are now evident linking internal cell bodies with the outer epithelial layer (Fig. 3E and F, arrows). The region at the anterior end, which will become the head capsule, is also more demarcated with contributing cells elongated along the antero-posterior axis (Fig. 3E). Behind this, an area in the centre of the body where structures are less well-defined, represents the developing neural ganglia (Fig. 3E, asterisk). By the time the germ ball has grown a stubby tail (body ∼80 μm, tail ∼50 μm, Fig. 2H), its cells are well organised, with some recognisable cercarial tissues. The anterior quarter is made up of cells that will become the head capsule (h, Fig. 3H). They are well ordered, but still with no recognisable structures at this stage. The second quarter contains the neural ganglia (Fig. 3H, asterisk), already visible as an anucleate mass. The third quarter contains the aggregated large acetabular gland-cells (a, Fig. 3H) in the centre and distinct lateral bands of cells on each side. The posterior quarter contains undifferentiated potential germinal cells, the “genital anlagen” of Cheng & Bier (1972). The cells in the tail are neatly arranged in lateral rows that will become the muscle fibres, and the tail is bifurcated from this early stage (Fig. 3H). There are still occasional contributing nuclei visible in the enveloping epithelium (Fig. 3H, inset).

### 3.3 Germ ball muscle development

**Fig. 4:**
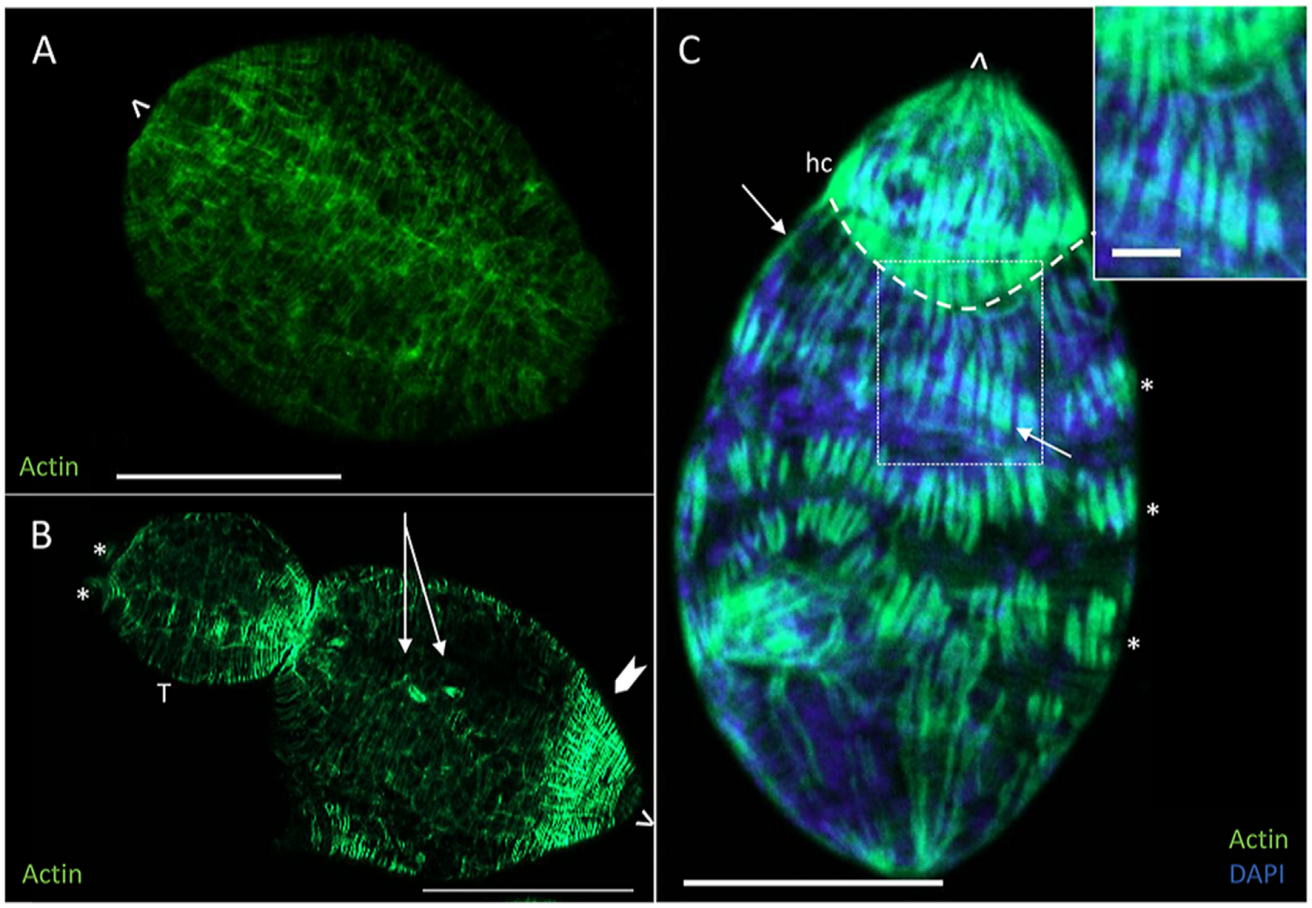
Germ ball muscle development revealed by phalloidin staining. **A** Flat-bottomed germ ball with circular actin filaments but no longitudinal fibres. **B** Germ ball with a stubby tail (T). Developing furcae (∗), longitudinal muscle at the anterior end (chevron), and flame cells (arrows) are indicated. **C** Maturing germ ball (tail detached during fixation) showing development of longitudinal muscle. Head capsule (hc) muscle boundary (white dashed curve), and bands of longitudinal muscle (∗) are highlighted. A diagnonal muscle fibre is indicated with an arrow at each end. Inset shows interdigitations of muscle fibres. The anterior of the parasite is indicated by ˆ. *Scale-bars*: **A**-**C**, 50 μm; inset, 10 μm.

Phalloidin staining of the smallest germ ball recovered after processing (75 μm long, Fig. 4A) shows that thin circular filaments of actin are present, and although some strands of longitudinal filaments are evident, they are far fewer in number. At the ‘stubby-tailedʼ stage, the outer circular muscle layer is denser in the region of the head capsule (Fig. 4B, chevron) than down the rest of the body. Fibres of actin can be seen in the tail (T, Fig. 4B), which has two small knobs where the furcae are starting to grow (Fig. 4B, asterisks). Two flame cells (Fig. 4B, arrows) are visible in the ‘stubby-tailedʼ germ ball (body ∼80 μm, tail ∼50 μm, Fig. 4B). Phalloidin staining of more developed germ ball (head ∼154 μm, Fig. 4C, tail detached during fixation) shows that the circular muscle layer of the body wall is formed first, with the underlying longitudinal muscles following. The latter are formed from bands of distinct myocytes (indicated by phalloidin staining of F-actin), arranged in circular tiers around the body, which differentiate after the head capsule muscle boundary (hc, dashed curved outline in Fig. 4C) has formed. The myofilaments of each longitudinal myocyte appear to be organised into pairs of myofibrils, and each myocyte extends until it meets and dovetails with those of the preceding and succeeding tier (Fig. 4C). In the illustrated specimen, this has occurred at the level of head capsule and at the posterior, while the three central bands (Fig. 4C, asterisks) are still clearly separate but aligned. Fine diagonal muscle strands are also visible at this stage (one such fibre is indicated by an arrow at each end in Fig. 4C).

### 3.4 The cercaria: gland-cells, alimentary tract and musculature

**Fig. 5:**
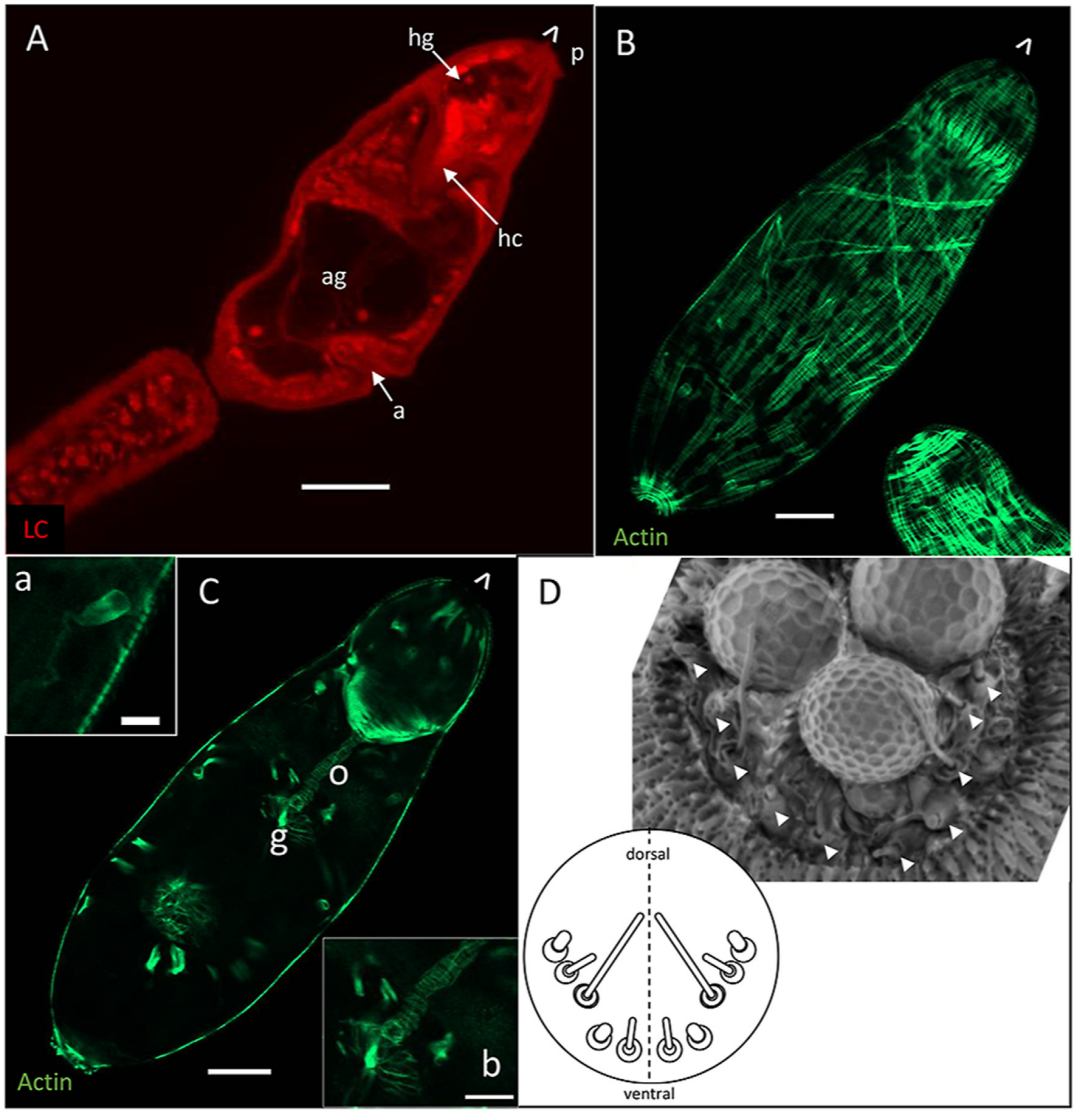
Morphology of mature cercaria. **A** Cercaria stained with Langeron’s carmine. Acetabular gland-cells (ag) now occupy a large volume of the cercarial body near the acetabulum (a). The head gland (hg) is visible within the head capsule (hc). **B** and **C**, Optical sections from a z-stack of a phalloidin-stained cercaria. **B** Peripheral slice showing longitudinal cells coalesced into continuous fibres, and transverse muscle fibres are present. **C** Central slice with the oesophagus (o) and gut (g) muscles. Inset **a** shows a flame cell from a different layer of the stack, inset **b** shows the circular fibres demarcating these organs. **D** TEM image of flattened apical area showing sensory endings (arrows), and a schematic diagram of their locations. The termini of three acetabular gland-cell ducts can be seen exuding secretory material. The image is provided, courtesy of Prof. James McKerrow and Dr Stephanie Hopkins (UCSF, San Fransisco). The anterior of the parasite is indicated by ˆ. *Scale-bars*: 20 μm.

The fully developed cercarial body (190 μm long, 40 μm wide) is covered with posterior facing spines, as is the tail. Most striking are the 10 now massive acetabular gland-cells which occupy approximately 1/3 to 1/2 of the cercarial body volume (Fig. 5A; Video 1), their ducts in two bundles extending to the front of the head capsule, where they reach the exterior of the larva passing through the tegument. There are numerous protrusions from the surface of the apical area (Fig. 5A, p; Video 1), which comprise the termini of the 10 acetabular gland-cells plus 10 ciliated sensory endings, arranged in a bilaterally symmetrical array (Fig. 5D). Morphologically, they comprise three types, distinguished by their protruding cilia: short (4), long (2), and short fat (4), presumably representing different sensory modalities. The head capsule, bounded by layers of circular and longitudinal muscle, especially intense on its posterior margin, encloses the much smaller head gland (hg, Fig. 5A). There are approximately 120 circular filaments in the cercarial body wall, but no information was obtained on the number of associated circular myocytes. The longitudinal muscles now form continuous strands running from anterior to posterior and anastomosing as they envelop the rounded posterior (Fig. 5B; Video 2); indeed, it is feasible that after fusion in the developing germ ball they are now a syncytium. Posterior to the head capsule there are also single muscle strands running diagonally across the body (Fig. 5B). The subterminal mouth on the ventral surface of the head capsule opens directly to the oesophagus, which is clearly demarcated by its own surrounding circular muscle fibres (Fig. 5C, o, inset; Video 3). The oesophagus connects to the short, bifurcated gut (Fig. 5C, g) which already has a complement of muscle fibres (Fig. 5C, inset; Video 3).

Phalloidin staining reveals eight flame cells in the cercarial body, and two proximal in the tail; they are arranged in pairs laterally (Video S2, Video S3). In the area between the acetabular gland-cells and the head capsule, other cells are small (∼2 μm in diameter) and tightly packed, whilst posterior to the acetabulum DAPI staining shows a cluster of undifferentiated cells on the ventral aspect, lying between the right and left post-acetabular gland-cells (Video 2).

### 3.5 Day 3 schistosomulum

**Fig. 6:**
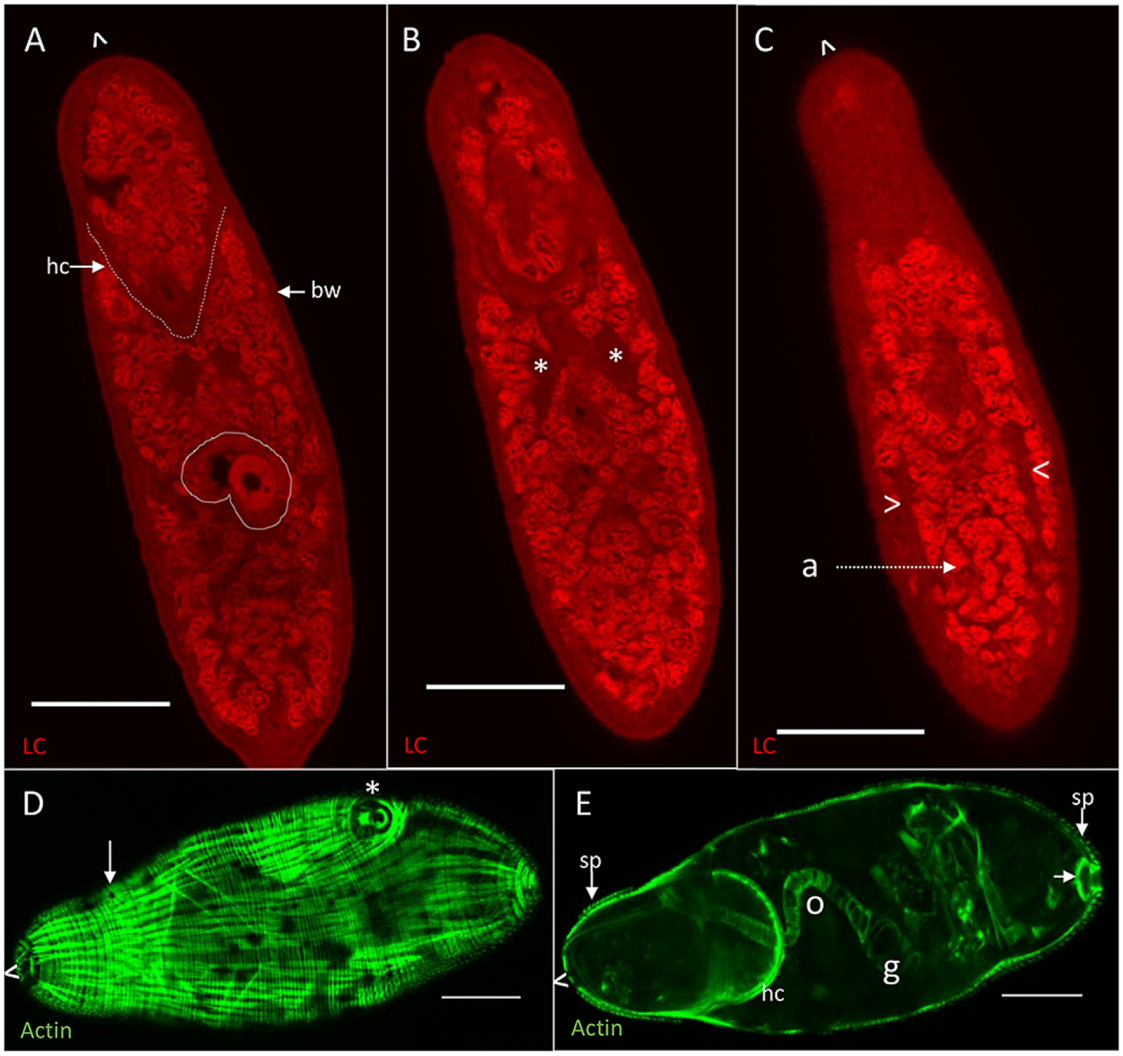
Day 3 schistosomulum. Optical sections from z-stacks of schistosomula stained with Langeron’s carmine (**A**-**C**) and phalloidin (**D**, **E**), which delineates only actin. **A** shows lack of acetabular gland-cells but still prominent head capsule (dotted outline, hc), the body wall (bw), and material present in the gut (white outline). **B** shows neural ganglia (asterisks). **C** shows the lateral nerve chords (chevrons) and ventral acetabulum (a). **D** Peripheral slice showing body wall muscle layers. The mouth (arrow) and ventral acetabulum (chevron) are visible. **E** Central slice showing the prominent posterior boundary of the head capsule (hc), the internal circular muscles surrounding the oesophagus (o) and gut (g), and bladder muscles (arrow). The anterior of the parasite is indicated by ˆ. *Scale-bars*: 20 μm.

The acetabular gland-cells and their ducts have completely disappeared in the day 3/4 skin schistosomulum (∼140 μm long, ∼55 μm wide) and the internal body space is filled by small largely undifferentiated cells (Fig. 6A). However, the head capsule (hc, Fig. 6A) with its strong muscle boundary, encompassing the head gland, is still intact. The body wall (bw, Fig. 6A) comprising tegument syncytium and underlying muscles appears as a thick but weakly stained layer. The gut is visible (Fig. 6A, white outline), with the lumen staining positive for protein. The anucleate mass of axons which comprise the paired neural ganglia (Fig. 6B, asterisks) are discernible, along with lateral nerve cords extending from them down the length of the body (Fig. 6C, chevrons). The nuclei of the ventral acetabulum form a discrete circular mass (Fig. 6C). Langeron’s carmine staining fails to detect any of the anterior sensory endings that were present in the cercaria (Fig. 5), indicating that they have been resorbed or shed.

Visualisation of actin in a peripheral slice (Fig. 6D) reveals the thin outer circular and stronger longitudinal muscle fibres, unchanged in appearance and distribution from the cercaria. The subterminal mouth and acetabulum are visible on the ventral surface in this superficial slice. Some supporting diagonal strands are also visible (Fig. 6D). Examination of an entire z-stack reveals the eight flame cells, positioned exactly as in the cercaria. The distribution of actin in a central slice (Fig. 6E) reveals multiple features including the thinness of the body wall muscle layer (∼1.5 μm). Intra-tegumental spines (sp, Fig. 6E) are also visible around the head region and posterior of the body but not the mid-region. The muscles of the excretory bladder wall (Fig. 6E, arrow) and pore are also visible at the posterior of the schistosomulum. Circular muscles delineate the oesophagus (o, Fig. 6E), longer than in the cercaria, running from the mouth (Fig. 6D, arrow) to the gut, which is larger, more prominent and positioned dorsally just anterior to the acetabulum (Fig. 6E).

### 3.6 Transcript and protein localisation

**Fig. 7:**
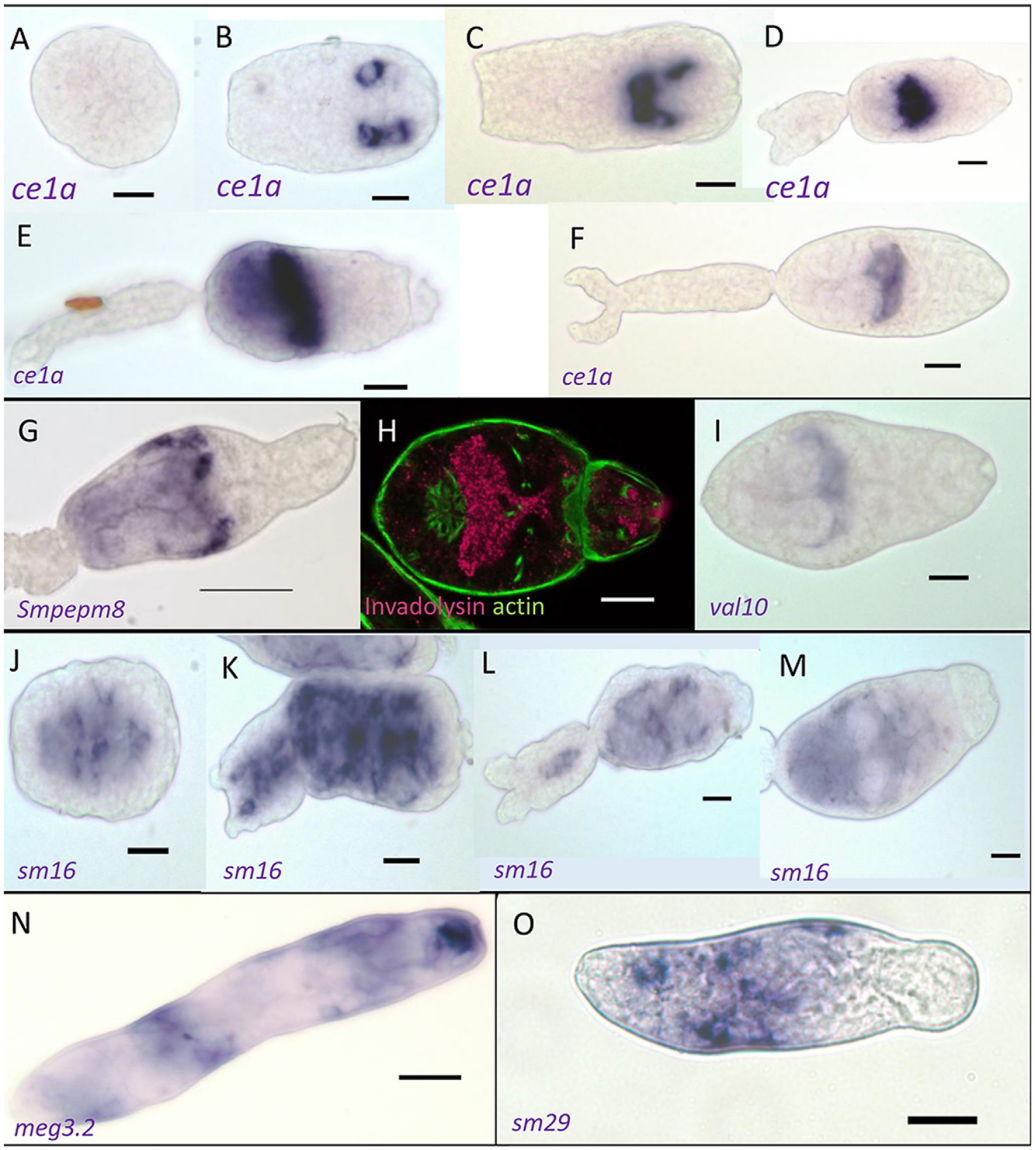
WISH and invadolysin protein distribution (anterior to the right). **A**-**F***Cercarial elastase* transcript (blue) in developing acetabular glands of germ balls from young spherical specimen (**A**) to nearly mature cercaria (**F**). **G***Invadolysin* transcript localised in the acetabular glands of a late germ ball. **H** ICC showing invadolysin protein in the acetabular gland-cells of a mature cercaria (tail detached during fixation). **I***VAL-10* is transcribed in the acetabular gland-cells of late germ balls (tail detached during fixation). **J**-**M***Sm16* transcript localised in germ balls. This transcript is produced throughout the body except the acetabular gland-cells, see clear patches in **J**, **L** and **M**. **N***Meg 3.2* transcribed in the head gland and putative tegument cell bodies of a day 7 schistosomulum. **O** Tegument surface marker *sm29* transcript localised in five to six tegument cell bodies of a day 3 schistosomulum. *Scale-bars*: A-F, 20 μm; N, 50 μm; O, 20 μm.

Parallel to characterisation by confocal microscopy of the morphological changes from germ ball to day 3 schistosomulum, we wanted to chart the expression pattern of genes encoding key proteins involved in the infection process (Curwen et al., 2006), using WISH. In two instances we were also able to examine the protein-staining pattern, using specific antibodies. One of the principal cercarial penetration enzymes, elastase 1a (*ce1a*), is transcribed in the acetabular gland-cells (Fig. 7A–F). Only the young spherical germ balls did not stain positive for *ce1a* mRNA (Fig. 7A). In the ‘flattened posterior’ germ ball (∼110 μm long), *ce1a* transcript is clearly discernible in the cytoplasm of acetabular gland-cells towards the anterior of the body (Fig. 7B). In a slightly older germ ball, with an extended posterior end, *ce1a* transcript is present in several cells – some are more posterior and some more anteriorly located (Fig. 7C). The early ‘stubby-tailed’ stage (Fig. 7D) exhibits the strongest staining. Here transcript is seen in a band across the middle of the body. The same pattern is seen in a germ ball at the ‘elongating-tail’ stage (∼100 μm long, Fig. 7E). By the time the germ ball is approaching maturity (body ∼125 μm long, Fig. 7F), staining intensity is reduced and appears as a narrow transverse band at the anterior of the acetabular gland-cell fundi.

In contrast, positive staining for the mRNA of a second penetration enzyme, invadolysin (*smpepm8*), is only seen in the most mature germ balls where it is detected at the anterior and lateral periphery of the acetabular gland-cells that are well developed by this stage (Fig. 7G). Germ balls at various stages of development from a small round germ ball, the ‘flattened-posterior’ stage, ‘stubby-tailedʼ specimen, and those with elongating tails were negative for this transcript (not shown). Invadolysin protein was also visualized in the cercaria by ICC, where it occupies the whole of the pre-acetabular gland-cell fundi and the ducts, extending through the head capsule to the apical area (Fig. 7H). Note that by this stage of development, the mRNA and protein have inverse distributions within the gland-cells, with mRNA indicating the site of protein synthesis at the anterior periphery (Fig. 7G) and protein storage everywhere else (Fig. 7H).

Three VALs have been identified in cercarial secretions and we chose VAL-10 as the exemplar. The expression pattern of VAL-10 (*val10*) mRNA is similar to that of invadolysin, described above (Fig. 7I). Expression of *val10* was not apparent in small round or ‘stubby-tailed’ germ balls (not shown). In nearly mature germ balls, staining is visible in a band across the middle of the body, along the anterior and lateral edges of the acetabular gland-cell fundi (Fig. 7I).

Sm16 protein has been proposed as a secreted immunomodulator but in stark contrast to the circumscribed patterns reported above for *ce1a*, *smpepm8* and *val10*, expression of its mRNA (*sm16*) is not limited to any particular tissue (Fig. 7J-M). It is first transcribed in germ balls with flattened posterior ends (Fig. 7J). Images of ‘stubby-tailedʼ germ balls reveal that *sm16* is distributed throughout the body, including the tail (Fig. 7K and L). However, there are patches that lack staining (Fig. 7J, L, M) and these correspond to the location of the acetabular gland-cell fundi and ducts. This pattern is most clearly evident in the ‘elongating-tail’ stage (Fig. 7M) but is also apparent in the ‘flattened-posterior’ and ‘stubby-tailed’ stages (Fig. 7J and N, respectively). In contrast to the above genes, where transcription ceases as the cercaria matures, members of the MEG-3 family increase in expression; these proteins are secreted by developing schistosomula. The expression pattern of MEG-3.2 mRNA (*meg3.2*) (Fig. 7N) is strongest within the head capsule, and weaker in circumscribed areas of the body, probably representing tegument cells. This conclusion is reinforced by the pattern of expression of *sm29* (Fig. 7O), encoding a well-authenticated tegument surface protein. Again, expression is confined to approximately five to six regions representing tegument cells.

### 3.7 Distribution of *smkk7* transcript and protein in larvae

**Fig. 8:**
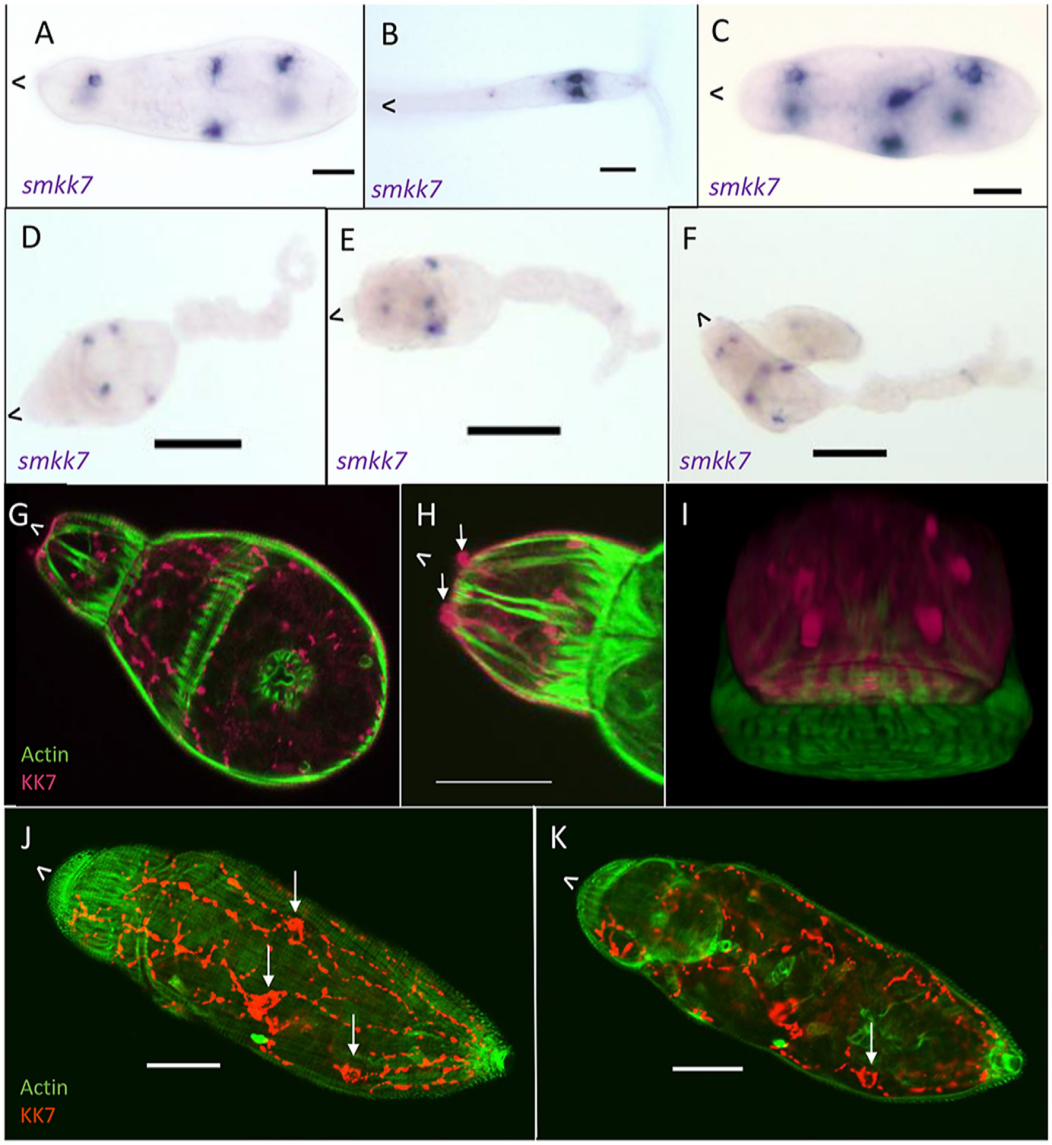
Localisation of SmKK7 in germ balls, cercariae and schistosomula. **A**-**F** SmKK7 mRNA localised by WISH using BM Purple as the substrate. **G**-**K** Distribution of SmKK7 protein detected by ICC (pink/orange) with phalloidin counterstain (green). **A** Three pairs of positive cells in the cercarial body. **B** Cercarial tail with two positive cells at the distal end of the tail. **C** The same pattern of positive cells in the day 7 schistosomulum. **D** Germ ball with central and posterior cell pairs positive for transcript. **E** Three pairs of positive cells present in the body. **F** Older germ ball with all three pairs of positive cells in the body, and a pair in the tail. **G** KK7 protein present as a network in the cercarial body. **H** Bulbous nerve endings (arrows) stain-positive at the anterior of the cercaria. **I***En face* view of cercarial apical region showing bulbous nerve endings. **J** and **K**, Optical sections taken from a z-stack of a day 3 schistosomulum with cell bodies (arrowed) and the dispersed network positive for SmKK7 protein. The anterior of the parasite is indicated by ˆ. *Scale-bars*: 20 μm except **D**–**F**: 50 μm.

SmKK7 protein was identified in the secretions of artificially transformed cercariae and so it might be anticipated that its mRNA (*smkk7*) expression would be like that of other secreted proteins. However, *smkk7* was highly localised in the germ ball, cercaria and schistosomulum stages (Fig. 8). In the cercaria, *smkk7* is transcribed only in six cells in the body (Fig. 8A) and two towards the posterior of the tail, just anterior to the bifurcation (Fig. 8B), not in the acetabular or head gland-cells. The same six-cell pattern is seen in the body of the day 7 schistosomulum (Fig. 8C). The *smkk7*-producing cells are arranged in pairs laterally; the anterior pair is within the head capsule, one pair is situated in the centre of the body, and the third pair is located towards the posterior. WISH in germ balls revealed that the central and posterior cells first transcribe *smkk7* at the ‘elongating-tail’ stage (Fig. 8D). This is followed by the appearance of the anterior (Fig. 8E) and tail pairs (Fig. 8F) as germ ball development proceeds.

In contrast to the sharply demarcated expression of *smkk7* mRNA, ICC revealed the presence of KK7 protein in a network of fine processes within the head capsule and throughout the cercarial body (Fig. 8G), which represents the peripheral nerve net (see Jamieson, 2016). This observation means that the *smkk7*-positive cells are the axon cell bodies. There are also KK7-positive knobs at the very anterior of the cercaria, external to the phalloidin-stained muscles (Fig. 8H); these are the bulbous sensory endings. A 3D-projection from a z-stack of the cercarial anterior reveals that these protuberances are arranged in three symmetrical pairs (Fig. 8I), which is thus consistent with the distribution of sensory nerve endings (Fig. 5D).

The pattern revealed by ICC in the schistosomulum is striking (Fig. 8J and K; Video 4). The same six cell bodies, identified by WISH are highlighted (3 visible in this optical section, arrowed), and the KK7 protein is distributed in a likely cyton network. Phalloidin counter-staining reveals that the central pair of SmKK7-positive cells lies just posterior and dorsal to the gut (Fig. 8K). Two processes extend from each of these cells to the anterior where the medial pair meet and join, dorsal to the oesophagus (Fig. 8K). Overall, the network comprises six ventral and six dorsal strands (Fig. 8J and K; Video 4) running along the body from the posterior towards the anterior; there are also some lateral connections (Video 4). In contrast to the cercarial body (Fig. 8G–I), SmKK7 protein is not discernible on the anterior surface of the schistosomulum (Fig. 8J and K) and the positive protruding knobs are no longer visible.

### 3.8 SmKK7 in adults

**Fig. 9:**
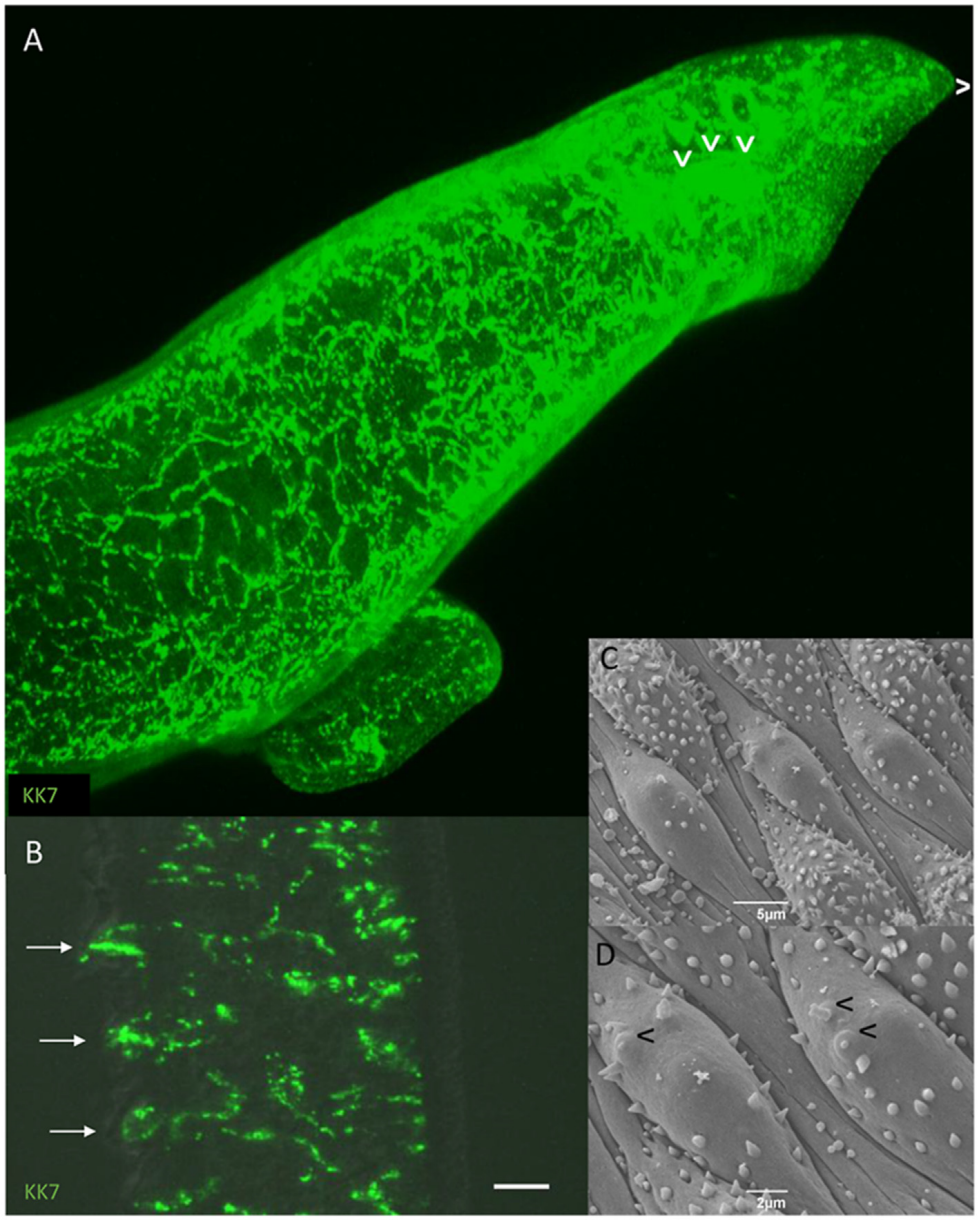
SmKK7 distribution in adult worms. **A** 3D-projection of a z-stack of an adult female worm after ICC for SmKK7 (green). Staining is especially dense at the anterior (chevron). **B** Projection of a z-stack of a section of an adult male after ICC for SmKK7 (green). The SmKK7 network extends into dorsal tubercles (arrows). *Scale-bar*: 10 μm. **C** and **D**, SEM images of dorsal tubercles showing alternation of spine-dense and sparsely-spined tubercles. Sensory endings are indicated with chevrons on the sparsely-spined tubercles only. The anterior of the parasite is indicated by ˆ.

As the SmKK7 protein proved to be a marker of the peripheral nervous system, we investigated its distribution in permeabilised adult worms. There, KK7 is visible as a distinct and fine meshwork beneath the body wall, revealing the complexity of the peripheral nerve net (Fig. 9A, Video 5). Staining is especially dense in the region of the body wall musculature and at the very anterior, around a structure that is probably the oesophagus (Fig. 9A, chevrons), the muscles of which have their own nerve net. The network projects outward into selected dorsal tubercles (Fig. 9B, arrows) of adult males where each branch terminates at the surface in a ciliated sensory ending (Video 6). It is notable that no similar pattern was observed in the tegument on the ventral surface, which lacks tubercles and is normally in contact with the female. Scanning electron micrographs reveal that rows of tubercles covered in spines alternate with tubercles bearing many fewer spines (Fig. 9C and D) The ciliated sensory endings are apparent only on the tubercles with fewer spines (Fig. 9D).

## 4 Discussion

Although the daughter sporocyst is a discrete generation and phenotype within the complex schistosome life-cycle, it is the least investigated stage, no doubt due to its inaccessible location within the hepatopancreas of the molluscan host. Indeed, life-cycle diagrams frequently ignore its existence or simply telescope it together with the mother or primary sporocyst derived by transformation from the penetrating miracidium (reviewed by Yoshino et al., 2016). Here we show that newly emerged daughter sporocysts can be recovered at day 21 post-infection, during their journey to the hepatopancreas, and analysed by confocal microscopy. The projecting spines on the anterior of the vermiform migrating stage undoubtedly allow it to gain purchase on and entry into the densely packed tissues of the hepatopancreas, where it becomes impossible to disentangle. The structures we have highlighted at the anterior of the vermiform stage may represent glandular tissue, but this would need conformation by transmission electron microscopy. Secretions from such a gland could contain lytic enzymes and/or natural detergents to aid the daughter sporocyst entry into the hepatopancreas.

The large germinal cells in the body of the migrating daughter sporocyst are in an undifferentiated state, undergoing active protein synthesis. Once *in situ* in the hepatopancreas, the daughter sporocyst body elongates and distinct brood chambers appear, where the cercariae develop from the germinal cells. The continuity of germinal cells in the asexual reproduction of intra-molluscan stages was confirmed by Wang et al. (2013). Totipotent stem cells in mother sporocysts produce progeny that independently initiate the embryogenesis of daughter sporocysts, which can then produce more daughter sporocysts or infective cercariae. This is a process of ‘polyembryony’, during which multiple embryos are produced from the same zygote with no intervening gamete production. A proportion of the germinal cells persist through to the adult worm where they form the primordia of the male and female gonads.

Germ ball development is an impressive process beginning with small balls of spherical cells showing minimal evidence of cellular differentiation at day 21 post-infection. The first fully-formed infective cercaria are detected ∼6 days later, suggesting that growth and differentiation is rapid. We imaged whole mount germ balls after removal from the daughter sporocyst, so their morphology was easier to observe. The head capsule and the thin enveloping tegument appear early, the posterior end of the germ ball flattens, and the tail begins to extend, with the bifurcated tip also apparent. Early ultrastructural studies (Hockley, 1972; Meuleman & Holzmann, 1975) revealed that a transitory primitive epithelium was replaced by a syncytial nucleated layer with ribosomes and mitochondria, which is the origin of the tegument that envelops the larval surface, as reported here. However, these nuclei disappear as connections arise to tegument cell bodies located beneath the muscle layers. There is evidence that the nuclei are lost by pyknosis, not extrusion (Hockley, 1972). This process happens first on the body and then on the tail, creating the arrangement of syncytial tegument connected to underlying cells, found in subsequent intra-mammalian stages. The cercarial tegument, bounded apically by a single membrane bilayer, is coated in a glycocalyx, needed for survival in fresh water, but it was not stained by the methods used here.

In the elongated cercarial body stage, distinct tissues begin to emerge, with the head capsule, central ganglia and acetabular gland-cells clearly demarcated first, while differentiation of the musculature appears quite late. The uniformly small outer circular fibres appear first. The images of myocytes participating in longitudinal fibre formation are very informative about the myogenic process. Individual nucleated myocytes, arranged in tiers around the body, align and fuse to generate the fibres of the body wall. We must assume that a similar process occurred previously with the circular fibres, but our images did not capture this. Diagonal muscle strands appear last. The process of muscle development begins at the anterior, with the head capsule muscle formation complete before myocytes appear in the tail, suggesting that a morphogenic gradient from anterior to posterior exists in the developing cercaria. The distribution of non-muscle actin in the barrel of flame cells and their associated tubules also allows the extent and layout of the excretory system in the different stages to be mapped.

The lifespan and infectivity of the free-swimming, non-feeding cercaria in fresh water is determined by the consumption rate of its glycogen stores (Lawson & Wilson, 1980). Its structure has been well described using both electron (Nuttman, 1971, 1974; Dorsey et al., 2002) and confocal microscopy (Collins et al., 2011). Of note for the current study are the possession of a primordial oesophagus and gut. Also crucial for host location and penetration are the numerous sensory papillae and the large, paired ganglia of the cercarial nervous system. As an aside, it is notable that similar structures were not observed in the daughter sporocyst but must exist to control the migration into the hepatopancreas. Six different types of sensory papillae have been documented in the cercaria, mostly concentrated at the anterior end (Dorsey et al., 2002). These include ciliated pits not detected here, which are postulated to have a chemoreceptor function, by analogy with other animals (Nuttman, 1971). Finally, as illustrated here, the cercaria possesses three types of gland-cell, the ten large pre-and post-acetabular gland-cells whose contents are expelled to facilitate skin penetration, and the much smaller head gland, housed within the head capsule. All three types of gland-cells release their contents at the flattened apical surface of the head.

We showed in a previous analysis of gene expression comparing germ balls, cercariae and day 3 schistosomula, that genes encoding the materials necessary for skin penetration are expressed early in development (Parker-Manuel et al., 2011). However, the genes encoding several cathepsins involved in nutrient digestion are also transcribed at this stage, so immediately after penetration of host skin, protein synthesis can begin. This theme of preparation for intra-mammalian life also applies to the tegument-cell body system, with associated genes including *sgtp4*, an *aqp-3*, and a *cd59* already transcribed in the cercaria (Parker-Manuel et al., 2011). The proteins ostensibly involved in skin penetration (elastase, invadolysin, Sm16, SmKK7 and VAL-10) are also germane to the current study. They were identified by proteomic analysis of secretions from artificially transforming cercariae (Knudsen et al., 2005; Curwen et al., 2006) and other approaches (Newport et al., 1988; Rao & Ramaswamy, 2000; Salter et al., 2002). The mRNAs encoding these proteins were localised using germ balls with developing acetabular gland between days 22 and 26 post-infection, as in our previous microarray study of the mammalian infection process (Parker-Manuel et al., 2011). We believe this is the first time that WISH has been applied to germ balls and required modification from our previous method (Dillon et al., 2007) because germ balls did not withstand treatment with proteinase K. In this respect, the ease with which germ balls disaggregate would appear to make them ideal candidates for single-cell sequencing.

Cercarial elastase (*ce1a*) expression was detected in the very early germ balls when the gland-cells were compact, almost certainly only in the six pre-acetabular gland-cells. As the cercaria develops the mRNA became localised around the anterior of each cell, confirming the observation that the protein synthesis machinery is pushed to the edge of the gland-cell fundi as the cells develop and fill with secretory vesicles (Dorsey, 1975). The invadolysin (*smpepm8*) and *val10* transcripts had a more circumscribed pattern around the periphery, similar to the late expression pattern of elastase. This could imply expression either late in cercarial development or a lower concentration of specific mRNA. Surprisingly, *sm1*6 mRNA did not follow this pattern, being expressed in almost all tissues, including the tail. The gene Smp_341790 encodes a signal peptide, implying that the Sm16 protein enters the secretory pathway, but the location in all cells and especially the tail suggests that it is not involved in skin penetration. Why was it found in the 0–3 h secretions by proteomics (Curwen et al., 2006)? Based on its detection in cercarial secretions, Sm16 had been implicated as an anti-inflammatory/immunomodulatory protein (Rao & Ramaswamy, 2000; Sanin & Mountford, 2015) modifying host dermal responses to facilitate parasite survival. These findings have recently been challenged by Berbardes et al. (2019). Sm16 was originally described as an analogue of vertebrate stathmin (Valle et al., 1999), which controls cell growth and differentiation through its capacity to regulate microtubule assembly. Our WISH images, showing gene expression in most cercarial tissues, point to an internal function for the Sm16 protein in schistosome cells. Perhaps these divergent views can be reconciled if we take account of the holocrine secretion of the acetabular gland-cells, where the entire cell contents are expelled into the host skin. Indeed, Sanin & Mountford (2015) found that the Sm16 protein was five times more abundant in the pelleted than the soluble fraction of the cercarial secretions; potentially sequestered in vesicles. Whether its presence in the acetabular gland-cell secretions is intentional, to downmodulate host dermal responses, or is simply a result of intrinsic schistosome cell biology, needs to be established.

The most remarkable observation from WISH on developing germ balls was localisation of the *smkk7* transcript in six discrete cells in the body and two in the tail. We initially eliminated the eight flame cells as a potential location. When we reacted permeabilised cercariae with specific antibody to SmKK7 protein it was immediately apparent that it localised not just to these six cell bodies, but also to a fine meshwork of filaments extending throughout the whole parasite: the only structure this could represent was the peripheral nerve net. In contrast, the mRNA was restricted solely to the axon cell bodies, which represent the site of SmKK7 protein synthesis. SmKK7 protein thus provides a marker for the peripheral nervous system, as confirmed by its presence within the bulbous sensory endings on the apical area of the cercaria. Since the *smkk7* transcript encodes a leader sequence, we infer that it passes through the endoplasmic reticulum and Golgi of the cell bodies to be packaged in vesicles for transport along the axons. It is thus a likely constituent of the numerous vesicles that pack the ciliated nerve endings of the cercaria (Nuttman, 1971; Dorsey et al., 2002).

Rapid entry into host skin is effected by the secretions of the acetabular gland-cells (Stirewalt & Dorsey, 1974; Paveley et al., 2009, Video S4), with ultrastructural studies of skin penetration showing their contents have been extruded *in vivo* by 2.5 h (Crabtree & Wilson, 1985). The process is accompanied by remodelling of the parasite, starting with loss of the tail and surface glycocalyx. This is followed by a shedding of the cercarial tegument plasma membrane and its instant replacement by the double outer bilayer configuration of the surface (Hockley & McLaren, 1973), which continues through to the adult worm (Wilson & Jones, 2021). It comprises a normal plasma membrane overlain by a secreted membranocalyx, the components of which are pre-packaged in the cell bodies of the cercarial tegument, ready for deployment (Skelly & Shoemaker, 2001). For example, schistosomula acquire the ability to take up glucose through the tegument, coincident with the glucose transporter SGTP4 appearing on the surface within 15 min after *in vitro* transformation and covering the whole body by 24 h (Skelly & Shoemaker, 1996). It is also notable that the intra-tegumental spines are lost from the mid-region of the body but retained at the anterior and posterior, to aid intra-vascular migration (Crabtree & Wilson, 1980).

The acetabular gland-cell remnants are quickly resorbed so that we detected no trace in the day 3 schistosomula. This and other remodelling processes may involve autophagy, poorly understood in schistosomes, although the transient appearance of autophagosomes has been reported, associated with removal of the spent acetabular gland-cells (Al-Adhami et al., 2005). In contrast, the fate of the head gland, located within the muscular head capsule, differs. Its secretions are utilised by the skin schistosomulum to aid penetration of dermal venules for onward migration (Crabtree & Wilson, 1985). The head capsule and gland are present at day 3 and appear to persist in the elongated migrating schistosomulum at least to day 10. The principal constituents of the secretions of this larval stage were identified by proteomics as a mixture of three MEG-3 proteins (DeMarco et al., 2010). Our WISH reveals the site of transcription of MEG-3.2 strongly in the head gland and weakly in putative tegument cell bodies. Diaz Soria et al. (2020) have also recently reported expression of *meg3* in the head capsule and tegument cell bodies of the day 2 schistosomulum. The function of the MEG-3 proteins is unclear, but they are also secreted by the mature egg when embolised in the intestinal vessels. The inference is that they interact in some way with the surrounding blood vessels (Wilson, 2012). The detection of tegument surface marker *sm29* in discrete cell bodies of the day 7 schistosomulum confirms both their distribution and approximate number.

The space freed up by acetabular gland-cell resorption allows the alimentary tract to expand. By three days, the elongated and wider gut pouches are located more posteriorly, just anterior to the acetabulum. The oesophagus has also elongated, but remains ∼4 μm wide, not large enough to admit erythrocytes. However, it appears that ingestion into the gut occurs (Thornhill et al., 2010) so plasma may well enter, since we detected material in the lumen. Indeed, our earlier study of gene expression patterns revealed mRNAs encoding MEGs associated with the oesophagus, and saposins plus cathepsins associated with the gastrodermis were up-regulated at day 3, compared to germ balls and cercariae (Parker-Manuel et al., 2011). Our previously published WISH data showed that MEG-4.2 transcript was expressed in the nascent alimentary tract at day 3 (DeMarco et al., 2010). The remainder of the body space is filled with numerous small undifferentiated cells, including a cluster of germinal cells at the posterior, but it is noteworthy that the schistosomulum does not increase in mass whilst it remains in the intravascular migratory phase (Lawson & Wilson, 1980).

The nervous system also undergoes significant remodelling between the cercaria and the skin stage larva, the most notable change being the loss of sensory endings at the extreme anterior; presumably their role in host location and penetration is now fulfilled. Their loss is interconnected with the identification of SmKK7 as a secreted protein since the *in vitro* transformation method used to trigger cercarial secretions results in rupture and release of sensory bulb contents. This does not seem to affect subsequent successful culture, or maturation if transferred *in vivo* (Harrop & Wilson, 1993). There must be a question as to whether SmKK7 has any role *in vivo* in skin penetration or its appearance secretions is just an artefact of technique. The central ganglia are also more prominent in the day 3 schistosomulum and the lateral nerve cords can be seen extending down the length of the body. The excretory system does not appear to change at this stage, being arranged as in the cercaria with six flame cells and their associated tubules in the same positions. However, the bladder (Thornhill et al., 2010) is more obvious along with a muscular pore, which is surrounded by circular and radial fibres.

SmKK7 was hypothesised to be a potassium channel blocker, on the basis of homology (34% identical; 52% positive, five conserved cysteine residues) with a venom protein (UniProt P59938; BmKK7) from the scorpion *Mesobuthus martensii* (Curwen et al., 2006). However, its localisation in the nerve net and at some sensory endings, discussed above, suggests a more likely function within the parasite’s nervous system. It has no close homologues in species other than parasitic flukes (*Paragonimus*, *Fasciola*, *Clonorchis* and *Opisthorchis*; NCBInr BLAST), not even the free-living planarian *Schmidtea*. Its limited species distribution suggests it may warrant further investigation as a drug target, given that disruption of nerve function has a debilitating effect on schistosomes (Parker-Manuel et al., 2015). It proved possible to knock down *kk7* expression in adult worms using RNAi but there is so much protein present along the nerve axons that no effect on phenotype was observed in the short term (Parker-Manuel, unpublished results). Interrogation of SchistoCyte (Wendt et al., 2021) reveals strong expression of *smkk7* in neural cluster 31, suggesting that this cluster represents peripheral nerve net cell bodies. Furthermore, WISH on adult worms confirms *smkk7* transcripts distributed in a random pattern over the whole body of adult males, consistent with its position in cell bodies of the peripheral nerve net (Supplementary Fig. 9a in Diaz Soria et al., 2020).

The study by Diaz Soria et al. (2020) subjected *in vitro* transformed 48 h-cultured schistosomula to enzymatic tissue disaggregation, followed by separation of live cells for sequencing of transcripts. A total of 15 clusters were identified and top markers for each were used to determine the tissue affinity of 13 (3 muscle, 4 neuron, 2 tegument, 1 oesophageal gland (*meg4+*), 2 parenchyma, 1 stem/germinal cell). This is far fewer than the 68 clusters obtained using the same approach with adult worms (Wendt et al., 2020). A notable omission is the saposins and hydrolytic proteases uniquely present in the “gut” cluster of adult worms (Wendt et al., 2020). Moreover, our microarray investigation of day 3 schistosomula (Parker-Manuel et al., 2011) showed that these genes were being actively transcribed. We conclude that the larval gastrodermal cells were not isolated by disaggregation of 48-h schistosomula. Conversely, multiple *meg* genes detected exclusively in the adult oesophageal gland-cell cluster were found in the larvae (Diaz Soria et al., 2020). Our study has shown that the tissues of the cercaria, and those of the schistosomulum which persist after transformation, begin to be formed in the very early germ balls. We reiterate the point that these intra-molluscan stages make ideal candidates for single cell sequencing to characterise the cells and tissues of this important life-cycle transition. The approach could identify e.g. gland-cell precursors, especially important for the post-acetabular gland-cells, where we have no authenticated markers.

## 5 Conclusions

Localisation of gene expression by WISH, and proteins by confocal microscopy, has allowed us to chart the increasing complexity of cellular organisation from germ ball to cercaria and then its transition by tissue remodelling into the skin-stage schistosomulum. We have followed the differentiation of myocytes as they give rise to the complex syncytial musculature of the body wall and head capsule. We observed that some transcripts for the secretory proteins involved in skin penetration are expressed in gland-cell precursors very early in germ ball development. However, those for *sm16* have a widespread distribution, while *smkk7* is confined to six cells within the body of the mature cercaria. In contrast, the SmKK7 protein is distributed throughout the peripheral nerve net and ciliated sensory endings; this distribution persists through to the extensive nerve net of the adult worm.

## Funding

Sophia Parker-Manuel was in receipt of a studentship from the Biotechnology and Biological Sciences Research Council (BBSRC). The funders had no role in study design, data collection and analysis, decision to publish, or preparation of the manuscript.

## Ethical approval

The procedures involving animals were carried out in accordance with the UK Animals (Scientific Procedures) Act 1986, as authorised on personal and project licences issued by the UK Home Office. The study protocol was approved by the Biology Department Ethical Review Committee at the University of York.

## CRediT author statement

Sophie Parker-Manuel: Data curation; investigation; visualization; writing - original draft and editing. R. Alan Wilson: Conceptualization; data curation, funding acquisition; methodology; project administration; resources; supervision; visualization; writing - original draft and editing. All authors read and approved the final manuscript.

## Declaration of competing interests

The authors declare that they have no known competing financial interests or personal relationships that could have appeared to influence the work reported in this paper.

## Appendix A Supplementary data

Video S1 Z-stack of a mature cercarial body (Fig. 4A), lying laterally with ventral surface at top, showing the volume occupied by the pre- and post-acetabular glands, the small head gland within the muscular head capsule and the protruding sensory endings of the apical area. Video S1

Video S2 Z-stack of a mature cercaria (Fig. 4B and C), ventral surface uppermost with mouth and ventral acetabulum visible, stained with phalloidin to reveal layout of body musculature; flame cell barrels and tubules are also detected, due to intracellular actin expression. Nuclei visualised with DAPI. Video S2

Video S3 Z-stack of a segment of the cercaria in Video 2 to illustrate the musculature of the alimentary tract. The 50% of the oesophagus that lies outside the head capsule has approximately 25 circular muscle filaments, plus two or three longitudinal filaments. The double sac of the gut at this stage is surrounded by a basket of filaments. A few longitudinal filaments run posteriorly from the gut basket, presumably to anchor it to other tissues. Flame cell barrels are also visible. Video S3

Video S4 Z-stack of a 3-day schistosomulum (Fig. 7J and K), dorsal surface uppermost, stained with phalloidin for actin distribution (green) and anti-KK7 antibody (red). The KK7 protein is concentrated in the individual cells bodies and distributed along the axons emerging from them to form the peripheral nerve net of the body and head capsule, located primarily beneath the muscles of the body wall. Video S4

Video S5 A 3D projection of the anterior of a female worm (Fig. 8A), permeabilised and stained with anti-SmKK7 antibody before imaging on a Zeiss Multiphoton microscopy. The extensive peripheral nerve net runs beneath the body wall and within oral and ventral suckers. There is also a net around the adult oesophagus. Video S5

Video S6 A 3D projection of a permeabilised adult male dorsal surface to show the relationship between the sub-muscular net and its branches passing up through the muscle layer to the dorsal tubercles where they terminate in ciliated sensory receptors. Video S6
